# Myocardial Infarction with Nonobstructive Coronary Arteries: A Diagnostic Challenge

**DOI:** 10.1055/s-0041-1728791

**Published:** 2021-06-16

**Authors:** Kofi Tekyi Asamoah

**Affiliations:** 1 National Cardiothoracic Centre, Korle Bu Teaching Hospital, Korle Bu, Accra, Ghana

**Keywords:** myocardial infarction, chest pain, atrial fibrillation, myocardial infarction with nonobstructive coronary arteries, cardioembolism, time in therapeutic range

## Abstract

Myocardial infarction with nonobstructive coronary arteries (MINOCA) is a significant cause of cardiovascular morbidity, especially among non-white women younger than 55 years. It is a working diagnosis that warrants further investigation due to its varied underlying pathophysiologic mechanisms. Investigations may be hampered by unavailability of testing modalities, cost, and the expertise to carry out the tests, as they are highly specialized. Clinical history is therefore important, especially in developing countries, to predict potential causes and institute empirical treatment without the luxury of tests. Some physicians are also unaware of this phenomenon and may dismiss symptoms as functional when a coronary angiogram shows nonobstructed coronary arteries, potentially resulting in patients suffering symptoms for longer and incurring extra cost. Most importantly, it leaves them at risk of major adverse cardiovascular events. This article presents a patient with atrial fibrillation who was diagnosed with MINOCA and highlights the diagnostic challenges in evaluating MINOCA.

## Introduction

Cardiovascular disease is the leading cause of death worldwide, 1 2 with 85% of events being due to myocardial infarction (MI) or stroke. 2 Among patients with MI, it has been found that up to 10 to 15% have normal coronary arteries on angiography. 1 3 4 5 6 This results in a diagnosis of MI with nonobstructive coronary arteries (MINOCA). MINOCA is a working diagnosis 3 4 5 6 7 8 with a predilection for younger non-white women (younger than 55 years) without traditional cardiovascular risk factors. 1 2 3 4 7 Diagnosis requires the following 2 4 6 8 :

- Clinical and biochemical signs of MI
- Coronary angiogram showing either normal coronary arteries or a lesion causing a <50% stenosis
- No other apparent clinical cause for the presentation.

While some studies report that patients with MINOCA are likely to be hypertensive 1 9 with lower rates of diabetes and dyslipidemia compared with MI with obstructive coronary artery disease (MICAD), Pasupathy et al stated no that there was significant difference between the two. 5 MINOCA is heterogenic and warrants further investigation. 1 2 3 5 6 7 Performance of these tests is limited by availability, cost, and expertise, 1 particularly in developing economies.

## Case Summary

A 42-year-old Ghanaian woman presented with a history of left-sided chest pain radiating to the ipsilateral shoulder, with associated dyspnea and diaphoresis for a few hours. The pain had subsided prior to her presentation at the hospital.

She was being managed with ramipril and amlodipine for hypertension and had had a metallic mitral valve replacement 12 years earlier for rheumatic mitral valve disease, following which she was anticoagulated with warfarin. She defaulted clinic reviews but returned following two short-lived episodes of sudden-onset aphasia 3 weeks apart, for which soluble aspirin was started. She was diagnosed with atrial fibrillation at this time and commenced on digoxin. It is unknown what her international normalized ratio (INR) was during that period. She was also started on cilostazol and atorvastatin a few years earlier on account of left femoropopliteal arterial disease confirmed by computed tomography angiogram following a period of intermittent claudication.

On examination, she had an irregularly irregular radial pulse of 54 beats per minute, with an apical rate of 78. Blood pressure was 161/99 mm Hg. The metallic mitral valve click was audible. All other clinical systems were unremarkable.

**Fig. 1:**
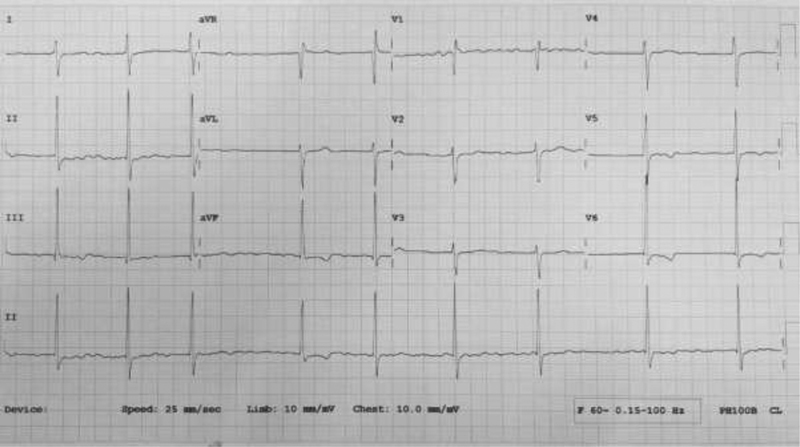
Electrocardiogram on presentation after chest pain.

Electrocardiogram (ECG) is shown in Fig. 1 and laboratory parameters are listed in Table 1 . Initial troponin I levels were more than 10 times the upper limit of normal ∼12 hours post onset of pain, dropping to approximately half the value over 48 hours. Echocardiogram showed a dilated left atrium with a functioning metallic mitral valve. Ejection fraction was 71%. There was no regional wall motion abnormality and she also had a mild aortic regurgitation.

**Table 1:** Results of laboratory investigations at presentation

Parameter	Value	Range
Hb	13.8 g/dL	11.5–16.5
MCV	90 fL	76–99
MCH	29.4 pg	26–34
MCHC	32.7 g/dL	30–37
Platelets	319 × 10 ^9^ /L	150–450
WBC	3.6 × 10 ^9^ /L	4–12
NEU	1.80 × 10 ^9^ /L	2–7.5
Lymph	1.50 × 10 ^9^ /L	1–4
CK-MB	9.53 ng/mL	<5
Troponin I	5.24 ng/mL	<0.5
Troponin I (48 h later)	2.43 ng/mL	<0.5
Sodium	139 mmol/L	136–145
Potassium	3.3 mmol/L	3.5–5.1
Chloride	101 mmol/L	98–107
Urea	3.1 mmol/L	2.1–7.1
Creatinine	75 µmol/L	44–80
AST	24.2 IU/L	<32
ALT	22.8 IU/L	<33
ALP	148 IU/L	96–279
GGT	36.8 IU/L	<38
T BIL	12.8 µmol/L	3.42–20.52
Dir BIL	9.2 µmol/L	<5
Total protein	85.2 g/L	64–83
Albumin	40.3 g/L	35–50
INR	2.1	
Total cholesterol	4.12 mmol/L	3.6–5.2
HDL	1.50 mmol/L	1.04–1.55
LDL	2.21 mmol/L	0–3.88
Triglycerides	0.91 mmol/L	0.3–1.71

Abbreviations: ALP, alkaline phosphatase; ALT, alanine transaminase; AST, aspartate aminotransferase; CK-MB, creatine kinase-MB; Dir BIL, direct bilirubin; GGT, gamma-glutamyltransferase; Hb, hemoglobin; HDL, high-density lipoprotein; INR, international normalized ratio; LDL, low-density lipoprotein; MCH, mean corpuscular hemoglobin; MCHC, mean corpuscular hemoglobin concentration; MCV, mean corpuscular volume; NEU, neutrophil; T BIL, total bilirubin; WBC, white blood cell.

A diagnosis of a non-ST elevation MI (NSTEMI) was made. Her drug regimen was maintained and an invasive coronary angiogram showed pristine epicardial coronary arteries. She was counseled on the need to adhere to her medications and review schedule, with more frequent INR checks.

## Discussion

MINOCA presents more frequently as NSTEMI, 2 4 7 with a modest rise in cardiac enzymes compared with STEMI. 4 Unfortunately, poor physician awareness results in missed diagnosis following a normal coronary angiogram, 8 leading to early discharge from clinic without appropriate investigation and management. 3 8 Such patients may incur significant financial costs through repeated hospital visits. 9

Atrial fibrillation is the commonest embolic cause of MINOCA 7 (accounting for up to 3% of cases 2 ) and an independent risk factor. 6 10 Patients with metallic mitral valves and atrial fibrillation are regarded high risk for cardioembolism, warranting a target INR of at least 3, whereas those with no risk factors have a target of 2.5. 11 Her INR at presentation was therefore subtherapeutic ( Table 1 ) and could have been responsible for the event. Her history of two previous aphasic episodes suggests possible cardioembolic transient ischemic attacks, which question her time in therapeutic range on anticoagulation.

The decision to request further testing for such a patient should be guided by clinical history and in limited-resource settings, by economic standing. It may not be economically expedient to pursue expensive diagnostic tests (even if available) which are unlikely to result in any modification of management particularly if symptoms are controlled, even if it gives diagnostic information. A carefully elicited history often reveals likely causes and risk factors such as substance abuse, atherosclerotic disease, and other comorbidities being suboptimally managed, which can aid in deciding on empirical therapy. This patient was already on the relevant medications due to her comorbidities. Such patients are likely to benefit more from good education on their diagnosis and the need for treatment adherence, in the author's view. Patients with no significant medical history will however require further investigation to help establish a diagnosis. Considering the patient was using warfarin, potential drug–drug and drug–food interactions will be vital in this patient's care and must specifically be addressed.

**Fig. 2:**
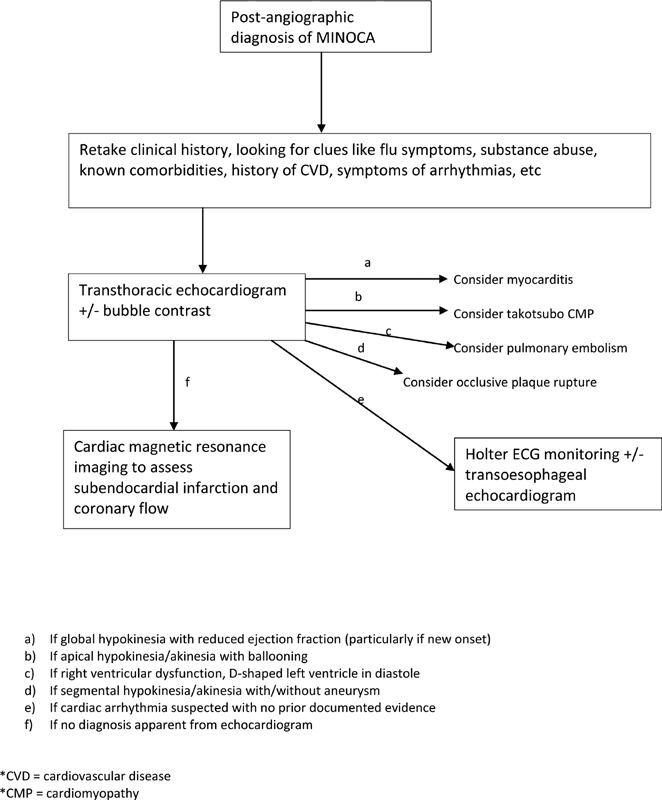
Proposed algorithm for investigating MINOCA in limited-resource settings. (a) If global hypokinesia with reduced ejection fraction (particularly if new onset). (b) If apical hypokinesia/akinesia with ballooning. (c) If right ventricular dysfunction, D-shaped left ventricle in diastole. (d) If segmental hypokinesia/akinesia with/without aneurysm. (e) If cardiac arrhythmia suspected with no prior documented evidence. (f) If no diagnosis apparent from echocardiogram. CMP, cardiomyopathy; CVD, cardiovascular disease; ECG, electrocardiogram; MINOCA, myocardial infarction with nonobstructive coronary arteries.

Specific diagnostic tests for various causes are listed in Table 2 and a proposed algorithm for investigation in limited-resource settings is detailed in Fig. 2 , to manage cost and free funds for other aspects of care. Many of the tests are quite complex and scarcely available in developing economies. Though expertise is limited to larger cities, transthoracic echocardiography is basic and should be performed in all cases. 12 This is the most available and least costly test and will give vital information on cardiac function and may suggest diagnoses such as pulmonary embolism, myocarditis, takotsubo cardiomyopathy, and cardioembolism. Transesophageal echocardiography, though largely unavailable, is useful if cardioembolism is suspected with no thrombus seen on transthoracic echocardiography. Prolonged Holter ECG monitoring in place of event recorders helps detect paroxysmal arrhythmias, as the latter are unavailable in many developing countries.

**Table 2:** Major mechanisms of MINOCA and relevant investigations for diagnosis

Mechanism		Diagnostic investigation
Epicardial vessels	Coronary vasospasm	Coronary reactivity testing /provocation testing, drug screening (e.g., cocaine)
	Spontaneous coronary dissection	Intravenous ultrasound, optical coherence testing
	Coronary plaque rupture	Intravenous ultrasound, optical coherence testing
Microvasculature	Coronary microvascular dysfunction	Coronary flow reserve
	Coronary embolism	Arrhythmia monitoring, echocardiogram (transthoracic, transesophageal), cardiac MRI, thrombophilia screen
	Coronary microvascular spasm	Coronary reactivity testing /provocation testing, drug screening (e.g., cocaine)
	Coronary slow flow	TIMI frame count

Abbreviations: MINOCA, myocardial infarction with nonobstructive coronary arteries; MRI, magnetic resonance imaging; TIMI, thrombolysis in myocardial infarction.

Cardiac magnetic resonance imaging is very useful, but a luxury for many. In Ghana, it is performed in just one center nationwide at a cost in excess of USD 500 and not covered by the government-based health insurance scheme. Some private health insurance schemes which require higher monthly premiums may help, though the premiums restrict access. Other helpful diagnostic tests such as intravascular ultrasound scanning, optical coherence tomography, and coronary reactivity testing with acetylcholine/adenosine/ergonovine are not widely available even in developed countries. Where available, steep costs limit accessibility, meaning patients may go undiagnosed and at risk of having further major adverse cardiovascular events.

All-cause 12-month mortality in MINOCA is at a significant 3 to 5%, 1 2 3 4 5 6 7 with similar functional and psychosocial limitations as MICAD. Prognosis is worse than single-vessel coronary artery disease (CAD) but more favorable than multivessel CAD. 4

## Conclusion

MINOCA causes significant morbidity and mortality, and it is vital that all physicians be aware of it, especially since it has a predilection for younger people. As challenging as it is to make a diagnosis, developing economies are further limited by availability and cost of tests. The clinical interview often gives useful information to assist in deciding on whether or not further investigations will have any impact on management decisions. Good physician education is required to ensure that such patients are well managed to get the best possible clinical outcome, while good patient education is important to prevent patient-dependent recurrence wherever possible. Public–private investment partnerships should be fostered to improve access to the needed investigative modalities for patients who may still require certain costly tests.
